# Location Isn’t Everything: Timing of Spawning Aggregations Optimizes Larval Replenishment

**DOI:** 10.1371/journal.pone.0130694

**Published:** 2015-06-23

**Authors:** Megan J. Donahue, Mandy Karnauskas, Carl Toews, Claire B. Paris

**Affiliations:** 1 Hawai‘i Institute of Marine Biology, University of Hawai‘i, Kāne‘ohe, Hawai‘i, United States of America; 2 Southeast Fisheries Science Center, Miami, FL, United States of America; 3 Department of Mathematics and Computer Science, University of Puget Sound, Tacoma, WA, United States of America; 4 Rosentiel School of Marine and Atmospheric Science, University of Miami, Miami, FL, United States of America; University of Auckland, NEW ZEALAND

## Abstract

Many species of reef fishes form large spawning aggregations that are highly predictable in space and time. Prior research has suggested that aggregating fish derive fitness benefits not just from mating at high density but, also, from oceanographic features of the spatial locations where aggregations occur. Using a probabilistic biophysical model of larval dispersal coupled to a fine resolution hydrodynamic model of the Florida Straits, we develop a stochastic landscape of larval fitness. Tracking virtual larvae from release to settlement and incorporating changes in larval behavior through ontogeny, we found that larval success was sensitive to the timing of spawning. Indeed, propagules released during the observed spawning period had higher larval success rates than those released outside the observed spawning period. In contrast, larval success rates were relatively insensitive to the spatial position of the release site. In addition, minimum (rather than mean) larval survival was maximized during the observed spawning period, indicating a reproductive strategy that minimizes the probability of recruitment failure. Given this landscape of larval fitness, we take an inverse optimization approach to define a biological objective function that reflects a tradeoff between the mean and variance of larval success in a temporally variable environment. Using this objective function, we suggest that the length of the spawning period can provide insight into the tradeoff between reproductive risk and reward.

## Introduction

Optimality theory has a long history in ecology addressing how the environment shapes behavior, energetic tradeoffs, and allocation of reproductive effort. Life history optimization is typically considered on an abstract environmental landscape and addresses the question: given the environment, what is the predicted optimal behavior, and does it match the observed behavior? As ecologists are better able to measure and model highly realistic fitness landscapes, an alternative approach—inverse optimization—becomes possible: given the observed behavior on a highly realistic fitness landscape, what is being optimized and what can it tell us about biological constraints?

Fish spawning aggregations are a striking example of synchronous reproductive allocation; some aggregations are highly predictable in space and time and have drawn particular attention from biologists and fishers [1,2]. Aggregating to spawn provides fitness benefits from reproducing at high density: aggregating may increase mate quality, increase fertilization success [3], and decrease the *per capita* risk of predation on gametes and larvae [4]. For the benefits of high density to accrue, a group of fish merely need to gather at the same place at the same time—requiring only a persistent landmark and temporal cue used for aggregation [5,6]. However, spawning locations may be more than a mere meeting place: the oceanographic features present at these specific locations and times may result in increased reproductive success [7,8]. The spatiotemporal fitness landscape of a spawning aggregation is the outcome of a complex interaction between larval behavior and oceanographic processes. Recent advances in high-resolution models of larval dispersal provide an opportunity to estimate the spatiotemporal fitness landscape of a spawning aggregation and examine the drivers of spawning aggregations as an inverse problem. We also consider the constraints on the optimization of this fitness landscape imposed by costs during other life-history stages.

**Fig 1:**
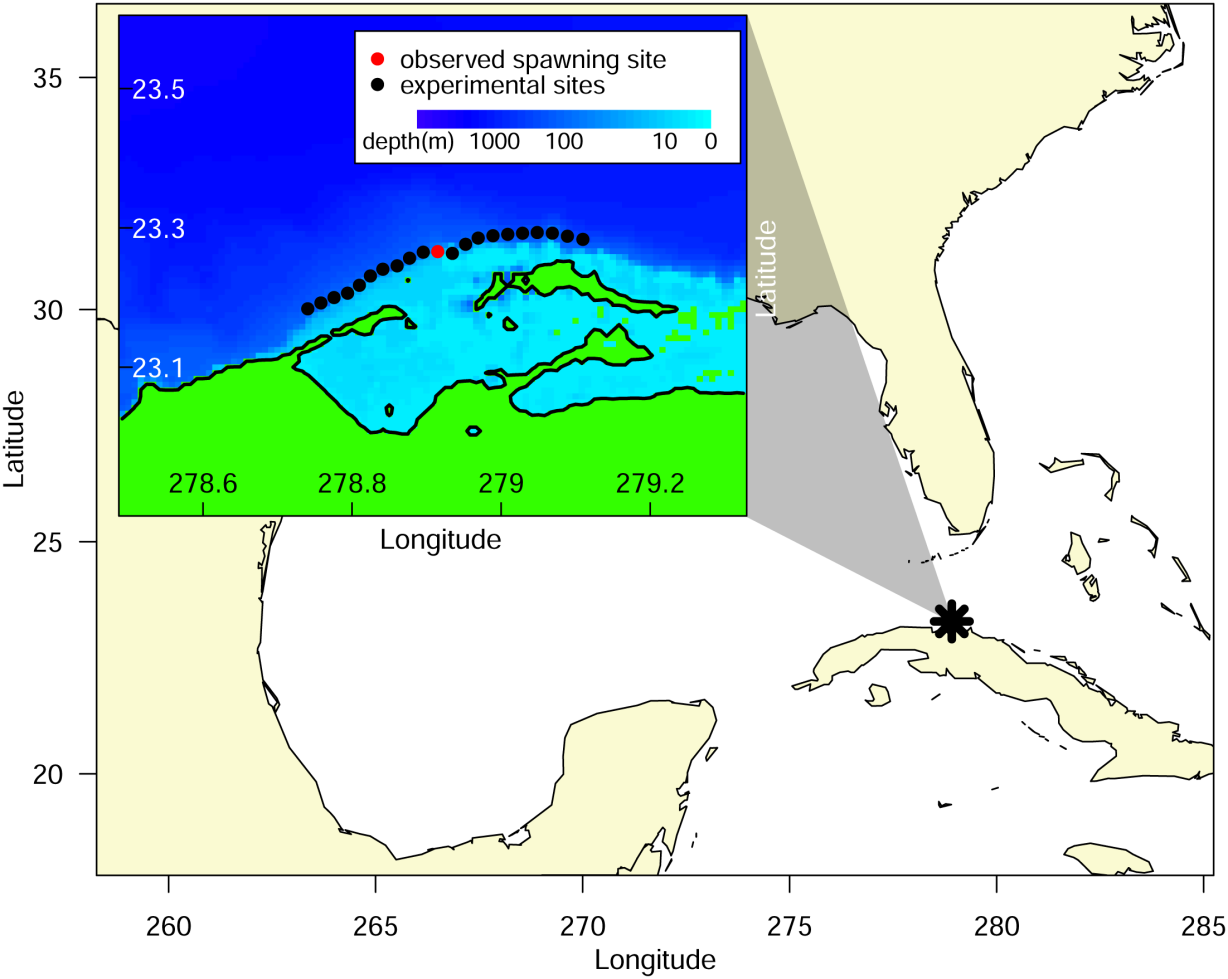
Spawning aggregation site for Lane snapper (*Lutjanus synagris*) in Punta Hicacos, Cuba. The inset shows the known spawning aggregation location (red dot) and the comparison locations (black dots) every 2 km to the north and south along the 25 m isobath.

We use a probabilistic biophysical model of larval dispersal that simulates individual life history traits and behavior (Connectivity Modeling System [9]; see Methods) coupled to a fine resolution hydrodynamic model (FLKeys-HYCOM 1/100 degree, (10)) to generate a reproductive fitness landscape as a function of the timing and location of the spawning aggregation of lane snapper (*Lutjanus synagris*) in Punta Hicacos, Cuba (Fig 1). We compared four measures of fitness (mean and minimum larval survival, age at settlement, and distance travelled) for larvae released on or adjacent to (i) the observed spawning days in the lunar month, (ii) observed spawning months in the year, and (iii) observed spawning location on the coast. On this stochastic fitness landscape describing the probability of larval success as a function of the spatial and temporal location of spawning release, we use an inverse optimization approach to understand the observed strategy of reproductive allocation by lane snapper and define a biological objective function in a spatially and temporally variable environment.

### Biophysical Model Results

**Fig 2:**
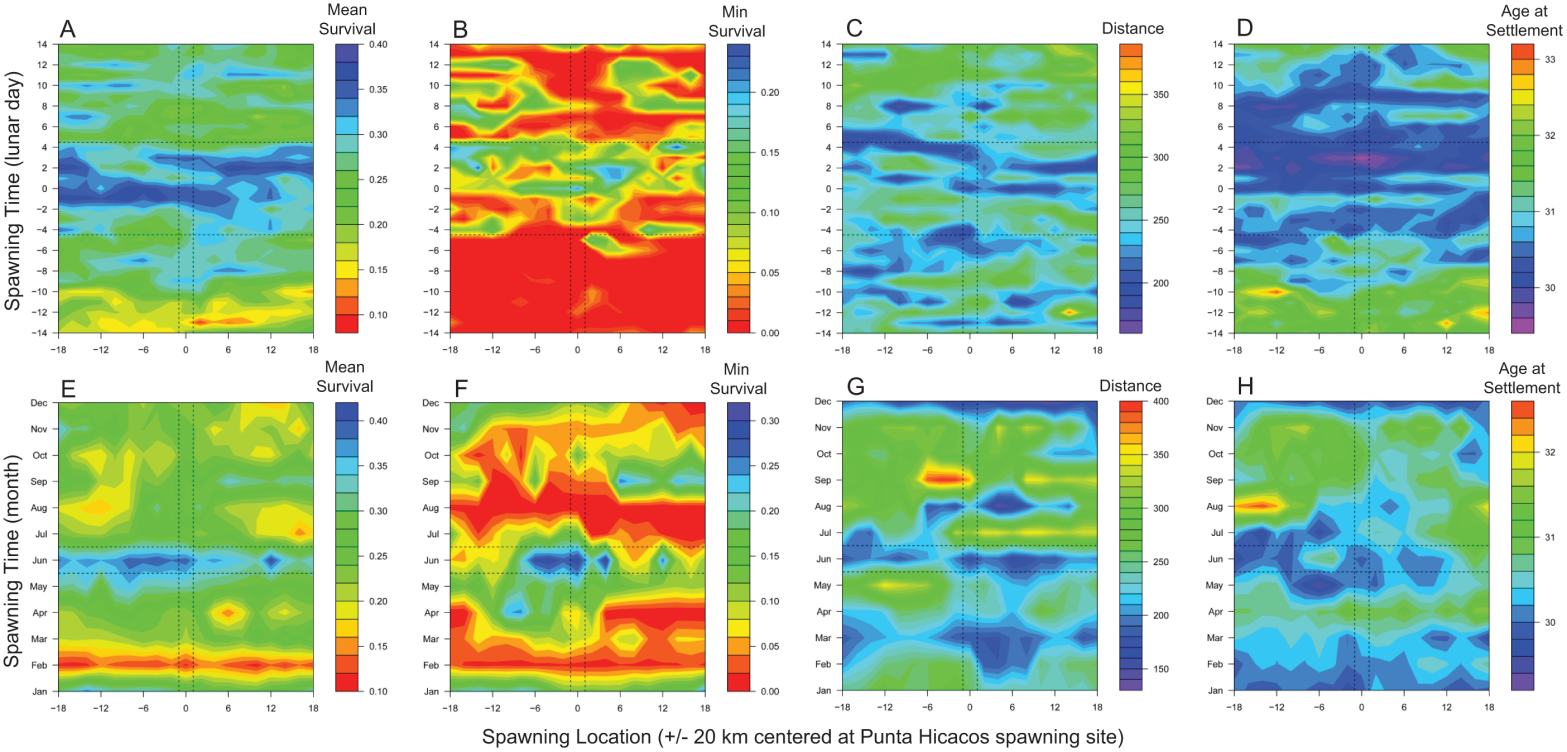
Metrics of larval success for spawning location (x-axis) and spawning time (y-axis). In the top row (A-D), the y-axis represents lunar days within a peak spawning month and, in the bottom row (E-H), the y-axis represents months within a year on the same lunar day. Larval success is measured as (A, E) mean larval survival, (B, F) minimum larval survival, (C, G) mean distance travelled between spawning and settlement, and (D, H) mean larval age at settlement. Color bars are given for each panel, and cool colors are associated with higher fitness (higher survival, shorter distance, younger age). Vertical dashed lines bracket the observed spawning location at Punta Hicacos; horizontal dashed lines bracket the observed spawning period.

Larval success was more sensitive to the timing than the location of spawning. Mean larval survival was higher within the observed spawning period than other days within the lunar cycle (Fig 2A,vertical axis), but mean survival was similar between the observed spawning location and adjacent locations (Fig 2A, horizontal axis). This pattern was even more striking for minimum larval survival (Fig 2B): the minimum percent larval survival over the ten simulated spawning periods was twice as high inside the observed spawning period compared to outside the observed spawning period (Table 1). Similarly, average distance traveled by settling larvae (Fig 2C) and larval age at settlement (Fig 2D) have stronger patterns along the temporal axis than the spatial axis, indicating greater sensitivity to timing than location.

**Table 1:** Metrics of larval success for observed and comparison spawning locations across lunar days and months.

	% Survival	Minimum % Survival	Age (days)	Distance Traveled (kms)
**Daily**				
Observed spawning location and lunar days	30.5% (1.3)	8.7% (2.2)	30.3 (0.2)	228.0 (8.2)
Adjacent spawning locations and lunar days	26.3% (0.2)	4.2% (0.2)	30.9 (0.02)	253.6 (1.3)
Lunar day and location of the maximum (survival) or minimum (age, distance) across all days/locations	-1 d, 8 km W	4 d, 14 km E	3 d, 0 km	8 d, 10 km W
**Monthly**				
Observed spawning location and peak months	38.5%	30.7%	29.9	168.1
Adjacent spawning locations and months	24.7% (0.4)	8.8% (0.5)	30.5 (0.04)	244.1 (3.5)
Month and location of the maximum (survival) or minimum (age, distance) across all months/locations	Jun, 12 km W	Jun, 0 km	May, 6 km W	Dec, 6 km E

Mean (+/- S.E.) values are listed for % larval survival, minimum % larval survival, larval age at settlement, and distance travelled from spawning to settlement. The mean values are compared between the observed spawning time (Daily: lunar days associated with peak spawning; Monthly: months associated with peak spawning) at the observed spawning locations *versus* times and locations not associated with (adjacent to) peak spawning. For monthly minimum % survival, the global optimum occurs in the observed month and location of spawning.

Comparing across months (Fig 2E–2H, Table 1), larval success was again more sensitive to the temporal scale than the location of spawning. Mean larval survival was highest in the observed spawning months (Fig 2E: highest mean survival between the dashed horizontal lines that bound the observed spawning months), but similar across simulated spawning locations (Fig 2E). Across months, the global maximum in minimum larval survival occurred during the observed peak month of spawning and at the observed spawning site (Fig 2F). Although more variable, successfully settled larvae from the observed spawning period also traveled shorter distances before settlement (Fig 2G) and settled at earlier ages (Fig 2H) than larvae spawned outside the observed period.

**Fig 3:**
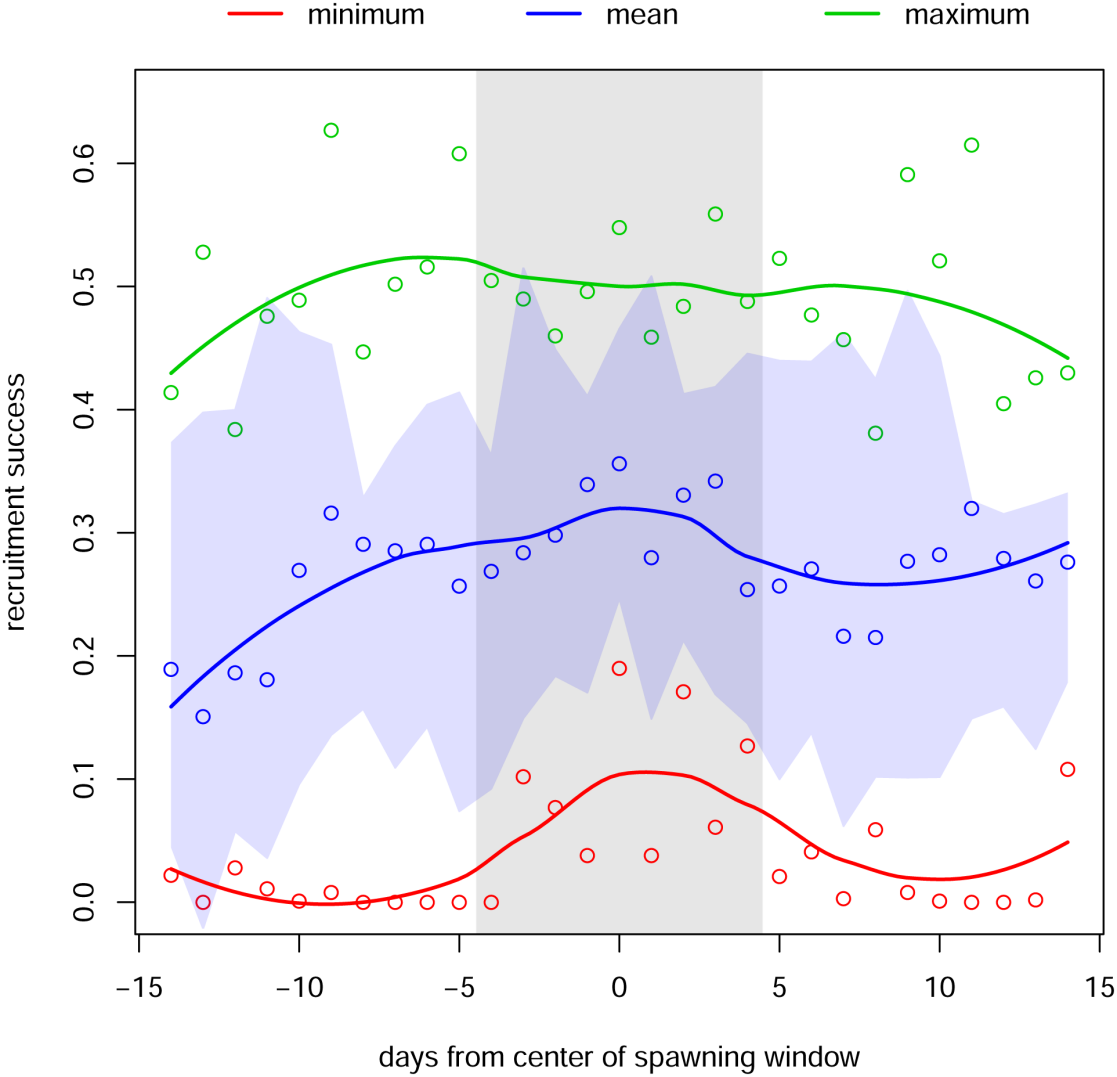
Maximum, mean, and minimum settlement success. Plotted values are calculated from 10 spawning periods (lunar months of May and June for 2003–2008). The vertical grey bar indicates the observed spawning period at Punta Hicacos. The horizontal blue swath portrays the variability in settlement success across the 10 spawning events (mean +/- 1 S.D.).

Overall, differences in reproductive success metrics across time (Fig 2, vertical axes) were more pronounced than differences across space (Fig 2, horizontal axes). Differences across time were important both at monthly scales (Fig 2E–2H) and at the scale of the lunar cycle day (Fig 2A–2D). Of the four metrics of larval success, mean survival and minimum survival both showed a clear pattern in differentiating observed spawning times from the comparison times (Fig 3).

**Fig 4:**
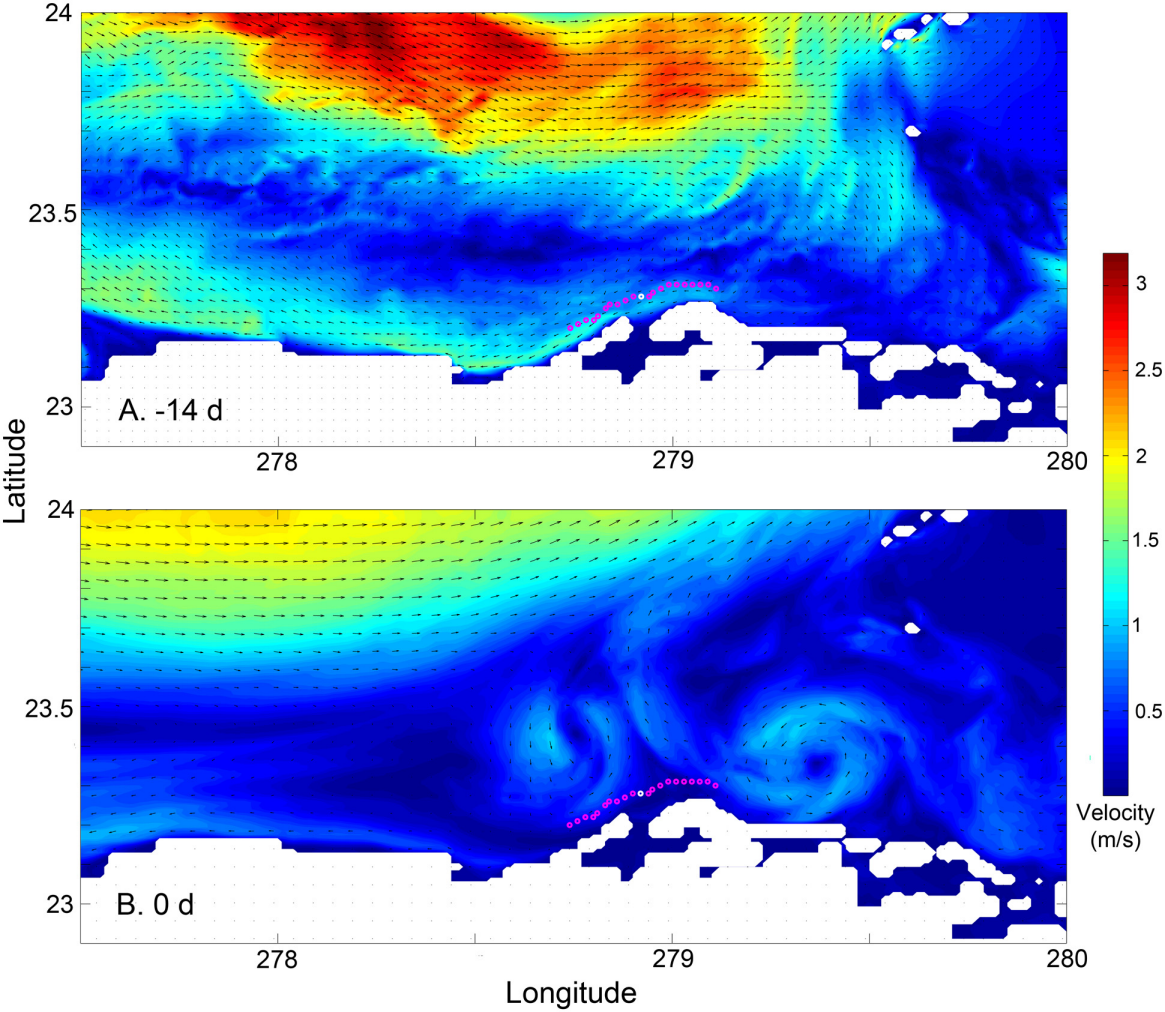
Oceanographic currents adjacent to Punta Hicacos (a) 14 days before the center of the spawning window and (b) on the central day in the spawning window. Dots along the coast indicate larval release locations; the central, white dot is a known spawning location and the adjacent red dots are the other release locations. The data for this plot is from 2004; similar changes are seen in other years.

**Fig 5:**
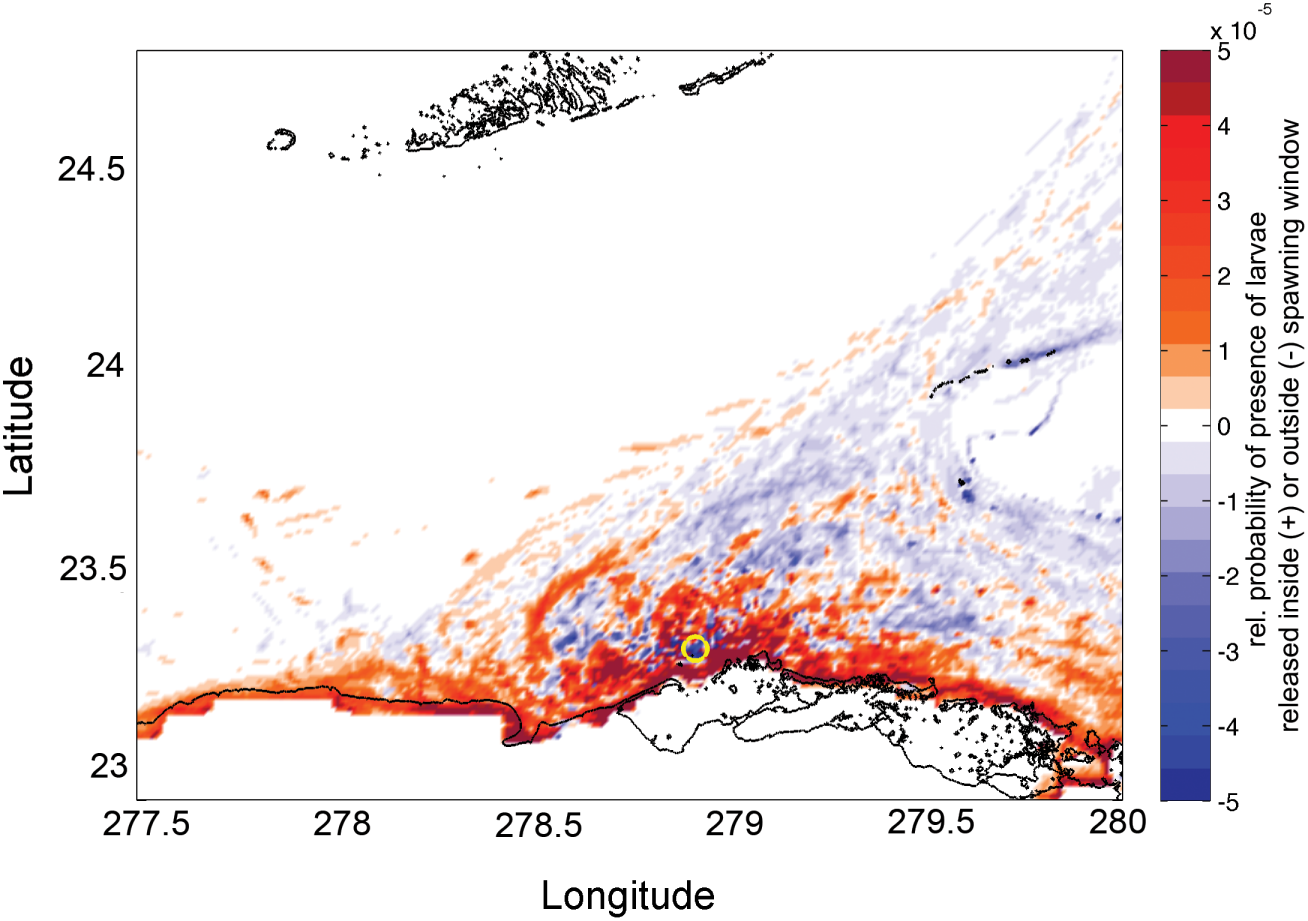
Difference in the dispersal kernels for larvae released inside and outside the spawning window. Color indicates the log of probability density of larval trajectories released inside and outside the spawning window; i.e., the probability that a larval particle released inside the spawning window was present in that grid cell minus the probability that a larval particle released outside the spawning window was present in that grid cell. A positive value (red) indicates that more larvae are present in the given grid cell when released within the spawning window. The yellow circle indicates the observed spawning location.

The mechanisms underlying these differences in larval success appear to be tied to mesoscale oceanographic conditions and their effects on larval dispersion. The proximity of the Florida Current to Punta Hicacos changes over the lunar month (Fig 4, S1 Fig), influencing the advection of larvae and larval success. Outside the spawning window, the fast moving Florida Current runs close to Punta Hicacos, advecting larvae away from the coast (Fig 4a). Within the spawning window, the northward shift of the Florida Current into the Straits generates eddies off Punta Hicacos (Fig 4b), which entrain larvae. The expected larval dispersal kernel (Fig 5) reflects the shorter duration and distance of larval trajectories inside the spawning window (Table 1) when advection by the Florida Current is weaker: more larvae released inside the spawning window remain close to shore and are retained near the point of release.

### Reproductive Allocation as an Inverse Problem

The simulated larval trajectories map the spatiotemporal spawning landscape onto metrics of reproductive success. While particular regions of this landscape are clearly associated with reproductive gains, an optimal reproductive strategy must balance these gains against risks within a stochastic environment. Our computational modeling approach allows us to analyze observed spawning strategies within a statistical framework and, thus, gain insight into how particular species navigate the tradeoff between risk and reward.

To illustrate the inverse approach, we use our computational results to inform a simple model that predicts the mean and the variance of reproductive return as a function of the length of the spawning window. The computational results that drive our model are summarized in Fig 3: (i) mean and minimum larval survival are maximal near the center of the temporal spawning window, and (ii) the variance of larval survival is relatively constant from day to day within the month. Under modest statistical and biological assumptions, these results suggest that the duration of the spawning window (i.e., the number of days that the aggregation spawns) represents a particular tradeoff between the mean and the variance of larval success, with shorter windows having higher mean returns but also higher variability. (See S1 Appendix for a detailed description of these assumptions, as well as derivations of the functional forms that follow.) Understanding this sort of tradeoff can shed light on a species’ resilience to reproductive failure and has potential implications for conservation.

A relatively generic way to cast this risk-reward tradeoff in the language of constrained optimization is to introduce an objective function of the form

$$\Gamma (m)\;=\;\mu (m)-\gamma \sigma^{2}(m),$$ (1)

where *μ*(*m*) and *σ* ^2^(*m*) are the mean and variance of the reproductive return associated with a spawning window of size *m*, and *γ* is a species-specific “reproductive resilience” parameter, with larger values of *γ* correspond to a lower tolerance to reproductive failure. Note that since the mean (i.e. the ‘reward’) makes a positive contribution to *Γ* and the variance (i.e. the ‘risk’) makes a (scaled) negative contribution, the optimal value of *m* maximizes *Γ*, although the value of *m* that does this will depend on the functional forms that define *μ*(*m*) and *σ* ^2^(*m*), as well as the parameter *γ*. (See S2 Fig for a geometric illustration of this dependence.) Objective functions of this form are common in portfolio management and have been used in life history theory to represent the mean-variance tradeoff [10–13].

**Fig 6:**
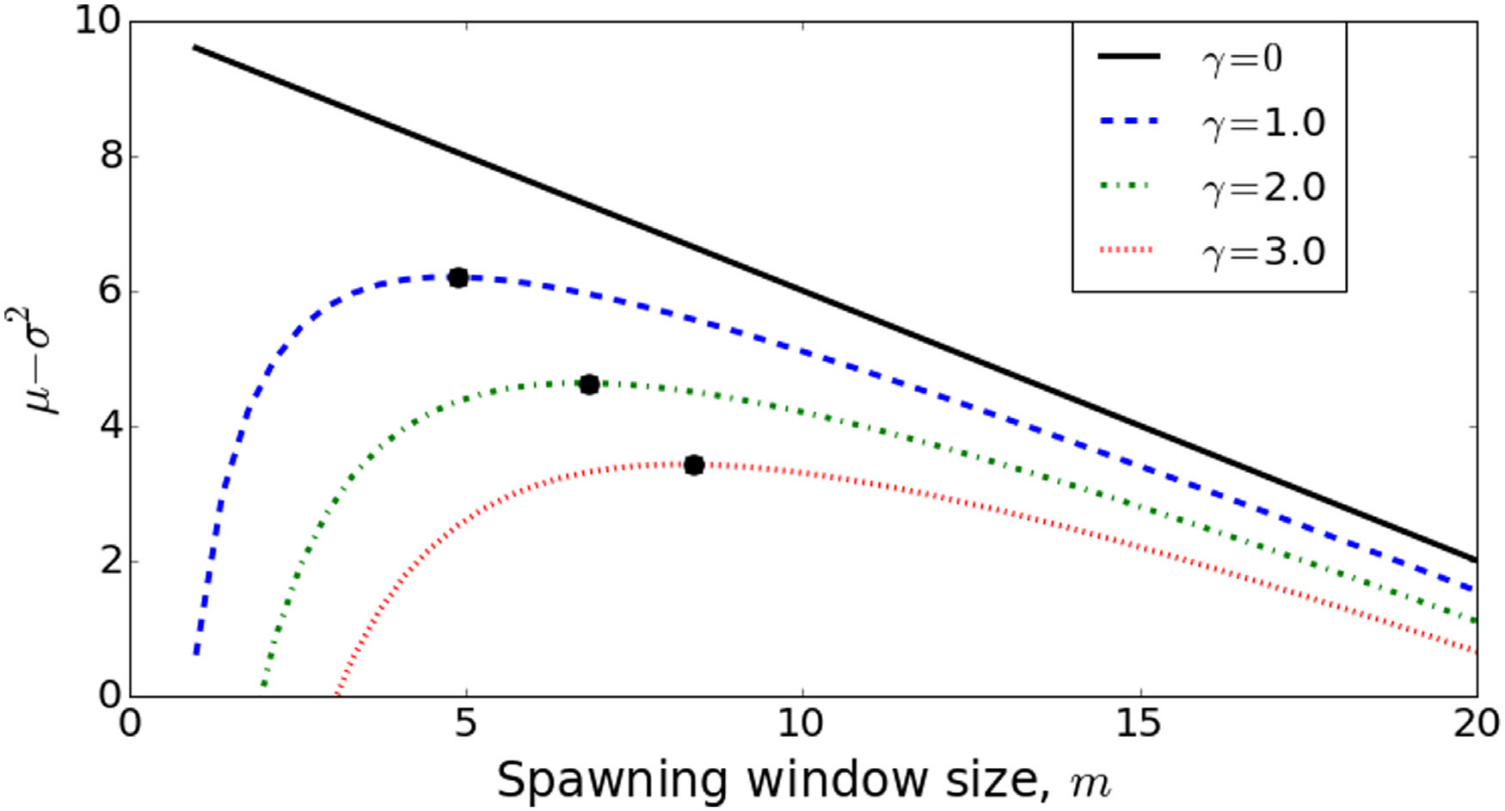
The objective function capturing mean-variance tradeoff, *Γ*(*m*) = *μ*(*m*)—*γσ* ^2^(*m*), plotted over a range of spawning window sizes, *m*, for three distinct values of reproductive resilience, *γ*. The local maxima represent the values of *m* that maximizes the objective. Note that each maximum occurs at a unique *m*, so if *m* can be observed, *γ* can be inferred. See S1 Appendix for further analysis of this figure. The functional forms for *μ*(*m*) and *σ*(*m*) are taken from S1 Appendix, Eqn. (A6), with parameters [*μ*_0_, *σ*, c] = [10, 3, 0.4].

In Fig 6, we examine this objective function for several levels of reproductive resilience. The functional forms that are used to produce these curves are derived in detail in S1 Appendix. Note that as reproductive risk tolerance decreases (larger *γ*), the optimal window size increases, and that there is a one-to-one correspondence between the value of *γ* and the position on the *m*-axis where the associated objective function is maximized. This correspondence allows us to infer the risk tolerance, *γ*, given that the size of spawning window can be observed and is assumed optimal.

This analysis is not limited to objectives of the form (1). In S3 Fig, we illustrate the implications of replacing the variance by the standard deviation, and, in S4 Fig, we introduce a one-parameter family of probabilistic objectives. S1 Appendix contains a mathematical derivation of all three approaches, as well as interpretive comparisons. As illustrated in Fig 6, however, the significant feature in all cases is that there is a one-to-one correspondence between parameters and the reproductive window size and, thus, in principle, a way to draw inferences about risk tolerance from the length of the reproductive window.

The potential strength of this analysis is largely comparative. Life-history theory suggests that species with longer reproductive lifespans will be more tolerant of reproductive failure and, therefore, have a shorter spawning window. However, comparisons may be complicated by species- or location-specific differences in the costs of migrating to and remaining on the spawning grounds. In addition, measuring the duration of the spawning window is challenging: fish often gather to the spawning site before spawning occurs, and individual fish may spawn different numbers of days. Despite these complexities, Punta Hicacos and other spawning locations in this region are shared by several species of snapper and grouper, providing a suite of interspecific and intersite comparisons for future study.

## Discussion

Prior studies have suggested that spawning aggregations may be located or timed to coincide with oceanographic events that are advantageous for the survival of eggs or larvae [4,7,8,14,15]. Gladstone (2007) found that drifters released at spawning aggregation sites moved more quickly offshore than drifters released at adjacent sites without aggregations and suggested that this more rapid offshore movement lowered predation risk [15]. In Little Cayman, Heppel et al (2008) found that drifters released the night of spawning were retained in eddies not present on nights prior to spawning and suggested that this retains larvae closer to suitable habitat [8]. Along the MesoAmerican Barrier Reef system, Karnauskas et al. (2011) found that eddies off promontories used by spawning aggregations were more predictable and had stronger potential vorticity than promontories without aggregations and suggested an increased potential for larval retention [7]. Here, simulated larval trajectories from gamete release to larval settlement indicated shorter larval duration, shorter distance travelled, and higher probability of success for larvae released in the observed spawning window. This difference in larval trajectories inside and outside the spawning window is driven by the position of the Florida Current; as the Florida Current moves offshore, nearshore eddies develop around Punta Hicacos, retaining larvae close to shore, and decreasing the probability of recruitment failure.

### Spatial and temporal variability in recruitment success

Our numerical results demonstrate that recruitment success is more sensitive to the timing of spawning than the spatial location, indicating that the precise location of spawning is less important, at least for the spatial scales and specific site considered in this study. However, recruitment success is just one component of overall fitness, and the spatial location of spawning may be important to an aspect of fitness that we did not consider, such as the energy needed to travel to the site or predation risk on adults at the aggregation. Lane snapper are primarily found in shallow reef lagoons and migrate to the shelf break during their reproductive window. They migrate to the spawning cape at sunset from the shelf break and come back the next day when they are spent. It is thought that portions of the aggregation alternate every other day to spawn during the 8–10 days around the full moon (R. Claro, *pers comm*). Notably, the observed spawning site is immediately seaward of a channel leading out of a nearby lagoon (Fig 1), i.e., the closest reef edge from lane snapper’s primary habitat (R. Claro, *pers comm*). Therefore, the observed spawning location may minimize travel cost or time exposed to increased predation risk for spawning lane snapper.

The relative importance of time versus space may also reflect our choice of scales: we examined the temporal variation in recruitment success over days and over months and the spatial variation in recruitment success at a resolution of 2 km. Temporally, the exploratory window of simulations was constrained by the number of days and months in the calendar year, and all possibilities were explored. Spatially, the scale of the numerical simulations was constrained at the low end by the grid size of the model (~1 km) and at the high end by the location of nearby spawning sites (~ 40 km away). The 20 km spatial scale also corresponds to the scale of eddies generated by the Florida Current, which are the likely driver of temporal variation. Thus, larvae released within several km of each other may be entrained in the same eddies and, therefore, subject to similar oceanographic processes.

### Management Implications

To the extent that the timing and placement of spawning aggregations are subject to refinement through natural selection, life history theory predicts that they should be “optimal” with respect to some (perhaps unknown) objective function. Our simulations suggest that the spatial and temporal location of spawning is more sensitive to minimizing recruitment failure than to maximizing mean larval success (Fig 3), indicating greater sensitivity to poor recruitment years than strong year classes. If existing spawning aggregations are fished out, spawning populations may form at new spatiotemporal locations that have lower reproductive success and are less optimal for population sustainability.

Our theoretical model refines our understanding further: it suggests that the length of the spawning window is a clue to a species-specific tradeoff between reproductive risk and reward. A quantitative understanding of these tradeoffs has the potential to refine policy objectives. For example, lane snapper shares spawning locations with other commercially important species (including mutton snapper, grey snapper, and cubera snapper [16]). Consistent differences in spawning window length between species can tell us about the relative reproductive resilience of these species, information that might help order conservation priorities and perhaps even flag tipping points in reproductive sustainability.

## Methods

### Lane Snapper

Lane snapper (*Lutjanus synagris*) is a commercially important, reef-associated fish with well-documented spawning aggregations throughout Cuba, which have declined in response to targeted fisheries [16]. We focused our study on the lane snapper spawning aggregation at Punta Hicacos in northwest Cuba (Fig 1) where the peak spawning aggregation occurs each year between the third quarter and full moon in May and June [16]. We used the Connectivity Modeling System (CMS, [9]) to model behaviorally realistic, stochastic larval trajectories from spawning to settlement. The CMS is an individual-based modeling platform that tracks individual larvae with species-specific life-history traits through nested coastal and large-scale physical oceanographic models on realistic landscapes from spawning to settlement. Successfully settling larvae are defined as particles traveling from spawning location to suitable settlement habitat, given the parameterized larval behavior, within the range of days defined by the pelagic larval duration. We compared trajectories from larvae released at the observed Punta Hicacos aggregation site during the observed aggregation period to larvae released at adjacent spatial locations (+/- 20 km in 2 km increments, Fig 1, inset) on (i) adjacent days (+/- 14 days in the lunar cycle) in the peak spawning months (May and June) and (ii) on different months on the peak lunar days. Across spatiotemporal locations, we compared four metrics of reproductive success: mean larval survival, minimum larval survival, mean larval age at settlement, and mean distance traveled by settling larvae (Fig 2).

### Ocean velocity fields

The CMS allows for particles to be tracked seamlessly over a series of multiple nested grids, such that both coastal processes and large-scale oceanic processes are resolved efficiently. For the base model, we used the HYCOM + NCODA Global 1/12° Analysis, a freely available ocean model with daily velocity fields available from 2003–2008 (www.hycom.org). HYCOM is a hybrid isopycnal coordinate ocean model (i.e., isopycnal in the stratified open ocean, fixed 10 m-depths in the unstratified surface layers, and terrain-following in shallow coastal waters), while allows for optimal simulation of both coastal and open-ocean features simultaneously [17]. The model is data-assimilative, using real-time observations of the ocean’s surface via satellite altimetry, as well as vertical profile information from CTDs, the ARGO observation program, and other sources. Nested within the Global HYCOM model was a very high horizontal resolution FLKeys-HYCOM 1/100° (~ 1 km) Analysis model [18], the restricted domain of which encompasses the western portion of the north-central coast of Cuba.

### Spawning time and location

Because submesoscale processes are thought to be important to larval transport (e.g., [7]), we carried out the larval dispersal simulations from a single spawning site falling near the center of the available high-resolution model: the Punta Hicacos site offshore of north-central Cuba (23°18´N, 81°03´W). Here, the spawning behaviors of the study species have been relatively well-documented [16]. Lane snapper aggregate at the site each month from April to September from the quarter to the full moon. Peak spawning occurs during this lunar period in May and June. During the day, aggregations of ripe fish shelter in the shallow reefs and each evening they migrate out from the reef edge to the spawning cape at approximately 25–40 m depth, (R. Claro, *pers*. *comm*.).

### Vertical movement, settlement, and other simulated behaviors

In the CMS, vertical movements of dispersing particles are defined via a probability matrix, which specifies the distribution of particles in the water column throughout time. Time steps for the probability matrix are most logically defined by using different stages of larval metamorphosis (e.g., preflexion, postflexion), as lutjanids tend to shift in their vertical distributions with these changes. Probabilistic distributions of lane snapper larvae in the different stages were taken from a detailed two-year study of larval distributions across the Florida Straits, which yielded 153 lane snapper larval observations [19]. Spawning depth was estimated to be 25 m [16], and particles are moved from this depth towards the surface after release to simulate egg buoyancy. The pelagic larval duration is reported to be 28–40 d for lane snapper [20]; particles arriving at suitable habitat within this competency period are considered to have successfully settled. For this study, suitable settlement habitat was defined by 3202 coral reef habitat polygons were created using the Coral Reef Millenium Mapping Project [21] dataset overlaid by an 8 km x 8 km grid, via a process described in [22]. Seven of the polygons sitting along the northern shore of NW Cuba near the target spawning aggregation site were further segmented in 2 km sections, corresponding to the distance between the release locations To resolve sub-grid scale turbulent processes, we added a random component to the motion of the particles at each time step: a horizontal diffusivity of 1 m^2^ s^-1^ and a vertical diffusivity of 0.001 m^2^ s^-1^. A background mortality rate of 0.0173 day^-1^ was applied throughout the larval period, which results in 50% mortality by the end of the pelagic larval duration. The ‘avoidcoast’ algorithm of CMS was also employed, which prevents particles from getting stranded on complex coastal topographies [9].

### Experimental simulation setup

To consider whether observed spawning behavior was representative of some adaptive advantage associated with a particular place and time, we carried out simulations of gamete releases at both observed spawning times and locations, and at alternative temporal and spatial locations where the study species are not observed to carry out spawning. To consider the effect of spatial variability, we selected alternate experimental release sites along the same isobath as the observed spawning site, in 2-km increments, up to 20 km east and 20 km west of the spawning site (Fig 1). To consider the effect of temporal variability, we carried out experimental releases for days within the peak spawning ‘window’ (i.e., quarter to full moon in months of May and June) and days falling outside this window. Two types of temporal variation were considered: a) daily variation, where releases were carried out in all days of the lunar cycle in May and June, and b) monthly variation, where releases were carried out on the peak spawning day (defined as 4 days before the full moon, the center of the spawning window) on all months of the year.

Experimental releases were carried out for all combinations of spatial and temporal variation, for all five years of hydrodynamic model availability. For each combination, we simulated the release of 1000 particles, and the CMS tracked the location over time and the fate of each individual particle. The number of particles necessary to differentiate survival rates between space-time combinations was determined by sensitivity analysis: 1000 particles per combination allowed for an average of 262 surviving larva (maximum 644 larva) and only 3% of combinations with zero survivors. To understand whether spawning at the observed location and time represented some adaptive advantage, we considered four “success metrics,” which were calculated for each of the space × time combinations: (i) mean larval survival (average proportion of larvae that survive and arrive at suitable habitat during the competency period), (ii) minimum larval survival (minimum proportion of larvae that survive and arrive at suitable habitat during the competency period), (iii) mean larval age at settlement (average number of days between spawning and larval settlement to suitable habitat), and (iv) mean distance traveled by settling larvae (average distance traveled by successfully settling larvae).) The latter two metrics may be relevant fitness parameters because there is some evidence (e.g., [23,24]) that larvae settling at younger ages and closer to the release site may have higher survival rates in the early recruitment stages.

## Supporting Information

S1 Appendix Mathematical details of theoretical model. This appendix contains a detailed development of the underlying mathematical theory, and formulations of several possible modeling variations.
(PDF)

S1 Data Input and output files for simulations. This zip file contains six files: (i) daily_exp_releaseFile.txt and montly_exp_releaseFile.txt are the Connectivity Modeling System (CMS) release files for our daily and monthly simulations; (ii) expReleaseLocations.csv lists the polygon number, latitude, and longitude of the spawning locations used in this study; and (iii) confile_daily_exp.csv and confile_monthly_exp.csv are the daily and monthly connectivity output files from the CMS. The release and connectivity files are, respectively, the standard input and output files for the CMS, and further information about the structure of these files is given in the CMS documentation (Paris et al 2013). The connectivity files are the data analyzed for Figs 2 and 3 and Table 1; the release files give all of the information necessary to re-run the simulations.
(ZIP)

S1 Fig Oceanographic currents adjacent to Punta Hicacos. Panels display the oceanographic currents around the study site from 14 days before the center of the spawning window until the central day of the spawning window. The first and last panels are Fig 4 in the text. Dots along the coast indicate larval release locations: the central, white dot is a known spawning location and the adjacent red dots are the other release locations.
(PDF)

S2 Fig Geometric relationship between γ and the maximizer. The constrained optimum is at the point where the level lines of the objective function are tangent to the constraint curve. This plot shows optima corresponding to two different objective functions, i.e. two different values of γ in eq (1) from the main text. The constraint is the solid black line labeled “feasible set”—it represents the image of the function *g*(*m*) = (*μ*(*m*), *σ*(*m*)) as *m* varies continuously. This specific functional form is from See S1 Appendix, Eq. (A6), with parameters [*μ*_0_, *σ*, *c*] = [10, 3, 0.4]. Note that since the constraint is concave down and the level lines of the objectives are concave up, each value of γ yields a single optimum.
(PDF)

S3 FigReplacing variance by standard deviation in the objective function.This figure is a recasting of Fig 6 in the main text, but with the variance in eq (1) replaced by the standard deviation. Note that the positions of the local maxima are different in this plot than in Fig 6, illustrating how the particular values of the “risk penalty” parameter depend on the problem formulation. This formulation of the problem admits a probabilistic interpretation: the probability that *$\overset{-}{x\;}(m)$* lies above the dashed lines is fixed for each line.(PDF)

S4 FigA probabilistic objective for which the optimal solution is a minimizer.This plot investigates an objective of minimizing the probability that reproductive success rate falls below a fixed threshold, *∈*. The five lines represent five different such thresholds. Each point on a line represents the probability that the reproductive success rate $\overset{-}{x}(m($ falls short of the corresponding threshold, and the trajectory of the line illustrate how this probability changes as the size of the spawning window increases. The black dots indicate the spawning window size that minimizes the risk of falling below the threshold. Note that each dot corresponds to a different value of *m*, so if one knows *m*, one can infer the tolerance threshold *∈*.(PDF)
