# Supplementary material for: Location Isn’t Everything: Timing of Spawning Aggregations Optimizes Larval Replenishment
Source: PLoS One. 2015 Jun 23;10(6):e0130694. doi: 10.1371/journal.pone.0130694 (PMC4477890; doi:10.1371/journal.pone.0130694)
Supplement: S1 Appendix — This appendix contains a detailed development of the underlying mathematical theory, and formulations of several possible modeling variations. (PDF) [file pone.0130694.s001.pdf]

S1 Appendix: Mathematical details of theoretical model

The purpose of this appendix is to flesh out the mathematical details of the modeling results presented in the section "Reproductive allocation as an inverse problem." The development makes use of the following notation:

p_i the probability that a larva released on day i survives
m total number of days in the spawning window
N total number of larvae released (over the entire spawning window)
x_i total reproductive success rate on day i (a random variable)
μ_i mean of x_i
σ_i standard deviation of x_i
$\bar{x}(m)$ total reproductive success rate over window of size m (a random variable)
$\mu(m)$ mean of $\bar{x}(m)$
$\sigma(m)$ standard deviation of $\bar{x}(m)$

Assumptions

The model makes the following assumptions:

1. Larvae are released at a uniform rate over the entire spawning window, i.e. the total number of larvae released on day i is N/m.
2. The survival of a larva released on day i (for $i = 1 \cdots m$) is a binary random variable with parameter p_i (whence the total number of surviving larvae released on day i is binomial with parameters N/m and p_i).
3. N/m and p_i are large enough that the rates x_i are approximately Gaussian.
4. The x_i are uncorrelated.
5. The means μ_i decrease with i.
6. The standard deviations σ_i remain at a constant level, i.e. $\sigma_i = \sigma$ for some constant σ.

The first of these assumptions represents a simple first approximation to the problem of finite resource allocation. The second is justified on the grounds that larvae released on the same day in the same area should have similar probabilities of survival. While some statisticians propose hard "rules" about the circumstances under which binomial random variables can be approximated by Gaussians, the third assumption merely acknowledges that the accuracy of the approximation depends on the parameter values, and that the parameters that emerge from our computational work lie well within the bounds of most approximation heuristics. The fourth assumption is not essential but it simplifies the analysis. The fifth and sixth assumptions emerge from the computational work, which suggests that reproductive success is maximized near the center of the observed spawning window and that the variance of this success is relatively constant. The fifth assumption implies that the index $i = 1$ corresponds to the center of the

spawning window, and that higher values of the index represent days successively further removed from this center. This arrangement is for mathematical convenience and represents no irrecoverable loss of generality.

Risk and reward

Since $\bar{x}(m)$ can be expressed as

$$\bar{x}(m) = \frac{1}{m}\sum_{1}^{m} x_i$$

the mean total reproductive success is given by

$$\mu(m) = \frac{1}{m}\sum_{1}^{m} \mu_i \tag{A1}$$

which by assumption 5 clearly decreases with m. Using assumptions 4 and 6, it is easy to show that the standard deviation of the total reproductive success is

$$\sigma(m) = \frac{1}{\sqrt{m}}\sigma, \tag{A2}$$

which also decreases with m, as σ is constant. Interpreting $\mu(m)$ as the mean reproductive "reward" associated with a window of size m and $\sigma(m)$ as the "risk" associated with that window, these trends indicate that the size of the spawning window induces an intrinsic tradeoff between reproductive risk and reward.

As noted in the main text, one relatively generic way to cast this tradeoff in the language of constrained optimization is to introduce an objective function of the form

$$\Gamma(\mu, \sigma) = \mu - \gamma\sigma^2, \tag{A3}$$

where γ is a parameter that represents a species- and perhaps location-specific tolerance for risk. (The higher the value of γ, the lower the tolerance for risk.) Maximizing Γ corresponds to making μ as large as possible and σ as small as possible. The maximizing pair (μ, σ) must be biologically feasible, in this case by expanding or contracting the spawning window. In other words, we suppose that nature seeks to maximize (A3) over all pairs (μ, σ) that lie in the image of the function

$$g(m) = (\mu(m), \sigma(m)). \tag{A4}$$

Within this framework, the value of m that produces the maximizing pair is the size of the optimal spawning window.

The precise form of the constraint g could depend on many things, including species biology and oceanographic conditions. If the constraint is differentiable, utility is maximized at the point where its graph is tangent to a level curve of Γ, as illustrated in S2 Fig. Note that the position of this point will depend on the value of γ, but that since the level curves of Γ are concave up for each γ, there will be a unique minimizer as long as the graph of g is, e.g., concave down.

Functional forms

In principle, the form of $g(m)$ could be estimated from data. Since in this work data is sparse and the model is mostly notional, we give $g(m)$ a simple functional form by assuming that the means μ_i drop off linearly in i, i.e.

$$\mu_i = \mu_0 - ci, \tag{A5}$$

where μ_0 is the survival rate at the center of the spawning window and c is a constant. (Note that this assumption is clearly incorrect for large values of i, but for small values it can be a reasonable approximation.) Substituting (A5) into (A1), and then (A1) and (A2) into (A4), the constraint $g(m)$ becomes

$$g(m) = \left(\mu_0 - \frac{c(m-1)}{2}, \frac{\sigma}{\sqrt{m}}\right). \tag{A6}$$

Analytic formulations of this sort have several advantages. First, they enable graphical analysis of the sort illustrated in Fig. 6 and S2-S4 Figs. Second, they allow one to calculate the constrained optimum precisely (a feature that begs the usual caveats about over-reading analytical models.) For example, (A6) can be substituted into (A3), and the resulting expression used to solve either for the optimal m (if γ is known) or for the value of γ (if the actual and thus presumably optimal value of m is known.) Making the substitution, differentiating with respect to m, equating to 0 and solving for m or γ yields

$$m^* = \sigma\sqrt{\frac{2\gamma}{c}}$$

and

$$\gamma = \frac{m^{*2}\, c}{2\sigma^2},$$

respectively, where m^* is the optimizing value of m. Finally, if the statistical distribution of the random variable $\bar{x}(m)$ is known, these functional forms can be used to perform probability calculations, as illustrated below.

Interpretations and alternative approaches

The objective function given in (A3) represents a tradeoff between the mean and the variance of the reproductive "payoff," with variance penalized in proportion to the size of γ. Broadly speaking, higher values of γ correspond to less tolerance for uncertainty (risk), though as noted in the main text, γ needs to be interpreted in a comparative sense, across different species and spawning locations, rather than in any absolute sense. Indeed, it is useful to recognize that values of γ are scaled by our specific choice of objective. Were we to rewrite (A3) using σ instead of σ^2, i.e.

$$\hat{\Gamma}(\mu, \sigma) = \mu - \gamma\sigma \tag{A8}$$

then the size of γ would still indicate the extent to which nature penalizes risk, but the scaling would be different. Interpreting γ as a measure of natural risk aversion thus makes sense in a relative context, with the caveat that different formulations of the objective function will result in different numerical values for this parameter.

S3 Fig. illustrates this idea by recasting Fig. 6 in the main text with objective (A3) replaced by objective (A8). Note that once again, each curve has a unique global maximum, and that the peaks shift to the right as γ increases, but that the correspondence between the optimal *m* and γ has been altered. It is worth emphasizing that there is no "right" version of this figure, but that different forms might admit different interpretations. Here, for example, using (A8) allows us to make a probabilistic interpretation: since $\bar{x}(m)$ is assumed normal, the well-known "68-95-99.7" rule indicates that its value will lie within 1, 2, or 3 standard deviations of the mean 68, 95, or 99.7 percent of the time, respectively. In other words, the curves represent different *probability* thresholds, where for each *m*, the probability that the objective $\hat{\Gamma}(m)$ falls above the curve is constant.

For a more substantial variation, we also consider a probabilistic objective. From a population level standpoint, the goal of maximizing reward while minimizing risk is connected to the idea of minimizing instances of very *low* success. In this light, it is interesting to ask what value of *m* minimizes the probability that total reproductive success falls below some particular threshold ϵ, i.e. to solve

$$m^* = \min_m P(\bar{x}(m) < \epsilon) \quad \text{(A7)}$$

Under assumptions 2-4 above, $\bar{x}(m)$ is approximately normal, and under the linear model for the rate at which the μ_i decrease, its mean and standard deviation are given by the first and second components, respectively, of $g(m)$ in equation (A6) above. The probability calculation (A7) can be turned into one involving standard normal random variables (i.e. with mean 0 and variance 1) by "normalizing", i.e. subtracting the mean and dividing by the variance. Using this technique, the right hand side of (A7) becomes

$$P(\bar{x}(m) < \epsilon) = P(Z < \epsilon_m),$$

where Z is a standard normal and

$$\epsilon_m = \frac{\epsilon - \mu(m)}{\sigma(m)} = \frac{\sqrt{m}}{\sigma}\left([\epsilon - \mu_0] + \frac{c(m+1)}{2}\right).$$

These probabilities can be read off a standard normal probability table. S4 Fig.. plots these probabilities over a range of m for several values of ϵ. The black dots represent the minima. Note that, just as above, there is once again a one-to-one correspondence between *m* and the value of the risk tolerance parameter, in this case ϵ.

Comments and caveats

In a broad sense, the usefulness of Fig 6 and S3-S4 Figs. lies in their power as tools for "inverse optimality," i.e. inferring biological objectives from observed behavior and statistical features of the environment. The underlying premise of this approach is that the observed behavior has evolved as an "optimal" response to the environment. While the same premise animates the "forward problem", which starts with an objective and solves for behavior, the focus in the inverse approach is on understanding the evolutionary objectives themselves. This goal is clearly closely aligned with conservation objectives.

It bears emphasizing that the work described in this appendix is still largely notional. Indeed, we propose simple, one-parameter classes of objective functions for analytic tractability, but nature's true objectives may well be more complex. On the other hand, we would argue that even if our proposed objectives are simple, they have the potential to distinguish survival requirements across species and spawning locations in a way that has meaningful implications for conservation. Our approach thus promises to be a meaningful *comparative* tool, and as such, something that has the potential to help order conservation priorities. Understanding how robust and practical this tool might be will require further analysis, most likely in conjunction with detailed behavioral and environmental data.
