# Supplementary material for: Location Isn’t Everything: Timing of Spawning Aggregations Optimizes Larval Replenishment
Source: PLoS One. 2015 Jun 23;10(6):e0130694. doi: 10.1371/journal.pone.0130694 (PMC4477890; doi:10.1371/journal.pone.0130694)
Supplement: S1 Fig — Panels display the oceanographic currents around the study site from 14 days before the center of the spawning window until the central day of the spawning window. The first and last panels are Fig 4 in the text. Dots along the coast indicate larval release locations: the central, white dot is a known spawning location and the adjacent red dots are the other release locations. (PDF) [file pone.0130694.s003.pdf]

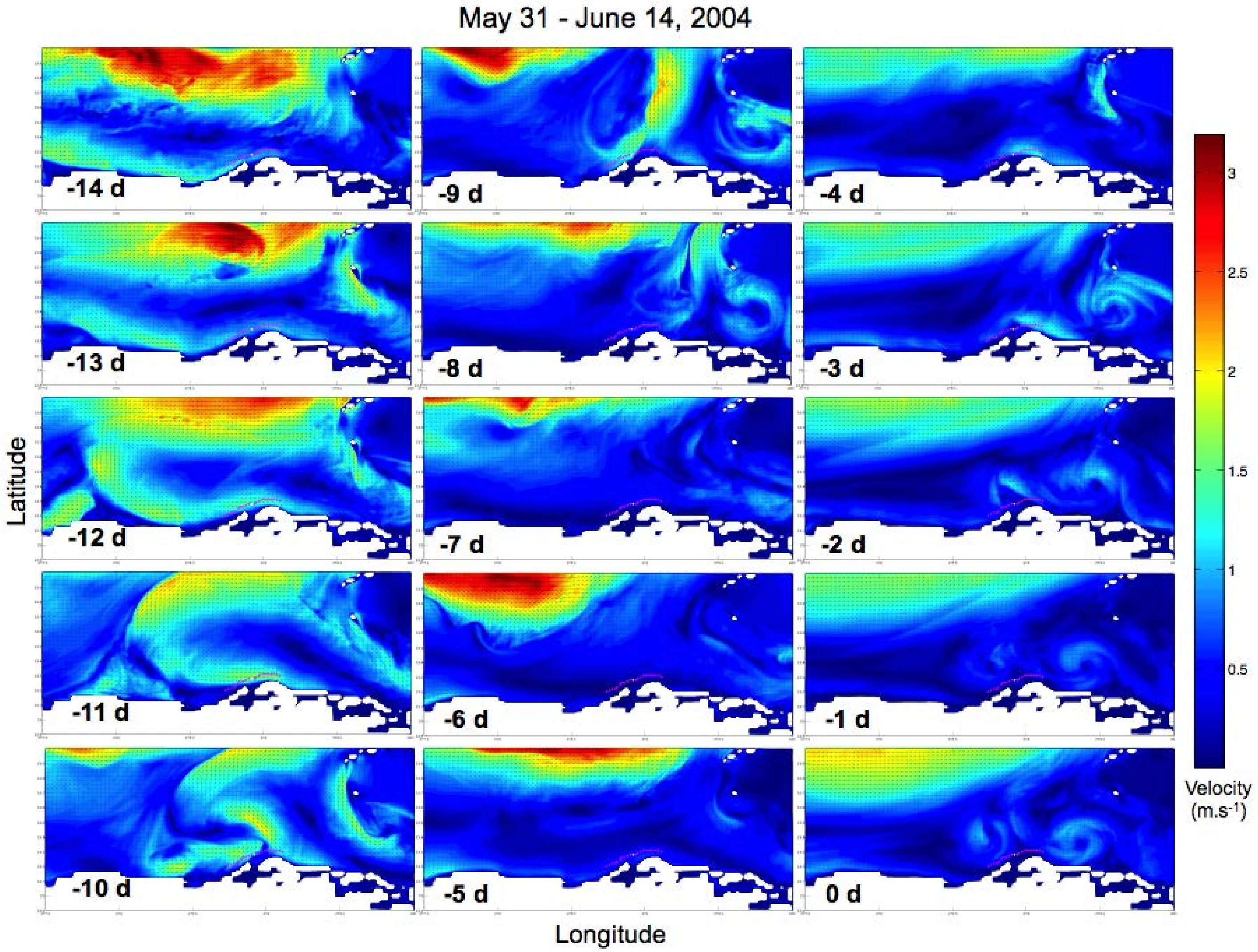

S1 Fig. Oceanographic currents adjacent to Punta Hicacos from 14 days before the center of the spawning window until the central day of the spawning window. The first and last panel are Figure 4 in the text. Dots along the coast indicate larval release locations: the central, white dot is a known spawning location and the adjacent red dots are the other release locations.
