# Supplementary material for: Location Isn’t Everything: Timing of Spawning Aggregations Optimizes Larval Replenishment
Source: PLoS One. 2015 Jun 23;10(6):e0130694. doi: 10.1371/journal.pone.0130694 (PMC4477890; doi:10.1371/journal.pone.0130694)
Supplement: S2 Fig — The constrained optimum is at the point where the level lines of the objective function are tangent to the constraint curve. This plot shows optima corresponding to two different objective functions, i.e. two different values of γ in eq (1) from the main text. The constraint is the solid black line labeled “feasible set”—it represents the image of the function g(m) = (μ(m), σ(m)) as m varies continuously. This specific functional form is from See S1 Appendix, Eq. (A6), with parameters [μ 0, σ, c] = [10, 3, 0.4]. Note that since the constraint is concave down and the level lines of the objectives are concave up, each value of γ yields a single optimum. (PDF) [file pone.0130694.s004.pdf]

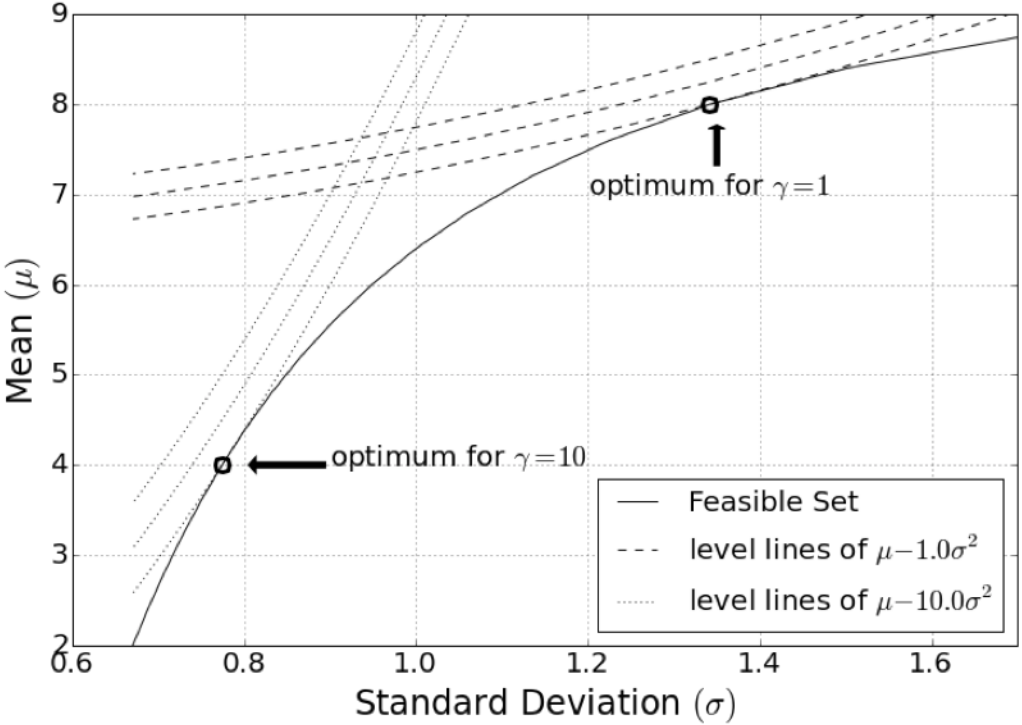

S2 Fig. Geometric relationship between the value of γ and the position of the constrained maximum. The constrained optimum is at the point where the level lines of the objective function are tangent to the constraint curve. This plot shows optima corresponding to two different objective functions, i.e. two different values of γ in equation (1) of the main text. The constraint is the solid black line labeled "feasible set"—it represents the image of the function $g(m) = (\mu(m), \sigma(m))$ as m varies continuously. This specific functional form is from S5 Appendix, Eq. (A6), with parameters $[\mu_0, \sigma, c]$ = [10, 3, 0.4]. Note that since the constraint is concave down and the level lines of the objectives are concave up, each value of γ yields a single optimum.
