# Supplementary material for: Location Isn’t Everything: Timing of Spawning Aggregations Optimizes Larval Replenishment
Source: PLoS One. 2015 Jun 23;10(6):e0130694. doi: 10.1371/journal.pone.0130694 (PMC4477890; doi:10.1371/journal.pone.0130694)
Supplement: S3 Fig — This figure is a recasting of Fig 6 in the main text, but with the variance in eq (1) replaced by the standard deviation. Note that the positions of the local maxima are different in this plot than in Fig 6, illustrating how the particular values of the “risk penalty” parameter depend on the problem formulation. This formulation of the problem admits a probabilistic interpretation: the probability that x-(m) lies above the dashed lines is fixed for each line. (PDF) [file pone.0130694.s005.pdf]

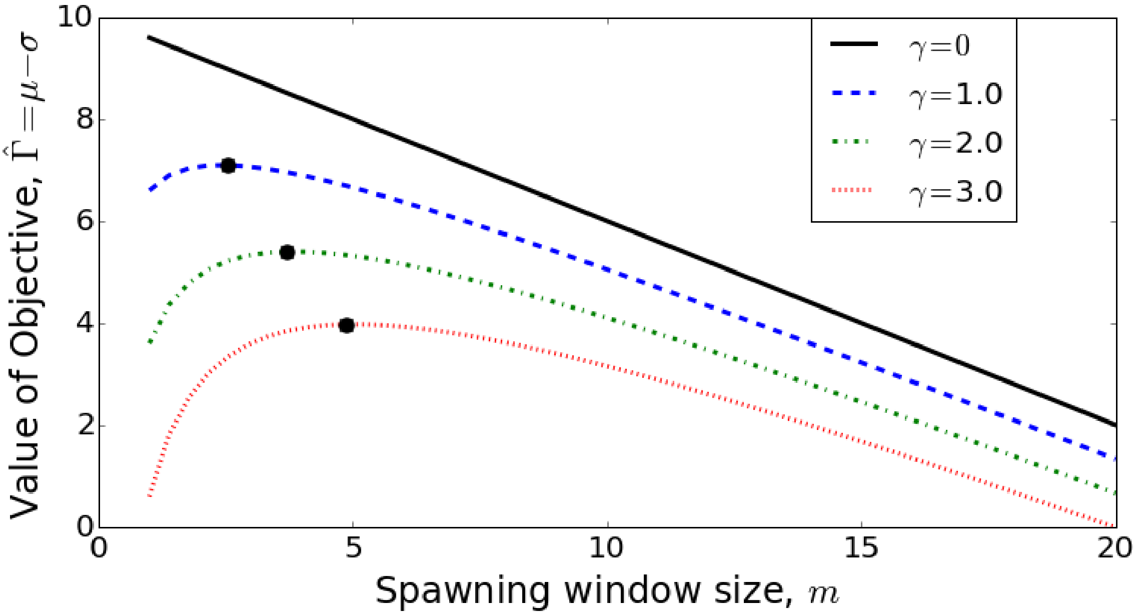

S3 Fig. Replacing variance by standard deviation in the objective function. This figure is a recasting of Fig. 6 in the main text, but with the variance in equation (1) replaced by the standard deviation. Note that the positions of the local maxima are different in this plot than in Fig. 6, illustrating how the particular values of the "risk penalty" parameter depend on the problem formulation. This formulation of the problem admits a probabilistic interpretation: the probability that $\bar{x}\,(m)$ lies above the dashed lines is fixed for each line.
