# Supplementary material for: Location Isn’t Everything: Timing of Spawning Aggregations Optimizes Larval Replenishment
Source: PLoS One. 2015 Jun 23;10(6):e0130694. doi: 10.1371/journal.pone.0130694 (PMC4477890; doi:10.1371/journal.pone.0130694)
Supplement: S4 Fig — This plot investigates an objective of minimizing the probability that reproductive success rate falls below a fixed threshold, ∈. The five lines represent five different such thresholds. Each point on a line represents the probability that the reproductive success rate x-(m( falls short of the corresponding threshold, and the trajectory of the line illustrate how this probability changes as the size of the spawning window increases. The black dots indicate the spawning window size that minimizes the risk of falling below the threshold. Note that each dot corresponds to a different value of m, so if one knows m, one can infer the tolerance threshold ∈. (PDF) [file pone.0130694.s006.pdf]

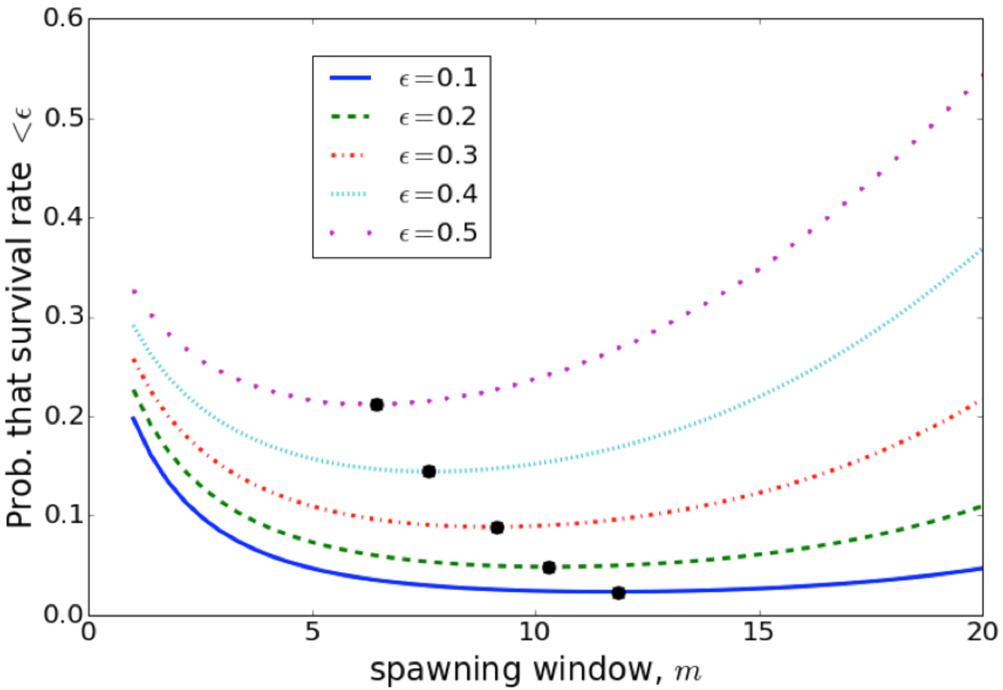

S4 Fig. A probabilistic objective for which the optimal solution is a minimizer. This plot investigates an objective of minimizing the probability that reproductive success rate falls below a fixed threshold, ϵ. The five lines represent five different such thresholds. Each point on a line represents the probability that the reproductive success rate $\bar{x}(m)$ falls short of the corresponding threshold, and the trajectory of the line illustrate how this probability changes as the size of the spawning window increases. The black dots indicate the spawning window size that minimizes the risk of falling below the threshold. Note that each dot corresponds to a different value of m, so if one knows m, one can infer the tolerance threshold ϵ.
